# The Role of E3s in Regulating Pluripotency of Embryonic Stem Cells and Induced Pluripotent Stem Cells

**DOI:** 10.3390/ijms22031168

**Published:** 2021-01-25

**Authors:** Yahong Wu, Weiwei Zhang

**Affiliations:** College of Life Sciences, Capital Normal University, Beijing 100048, China; 17835061377@163.com

**Keywords:** ubiquitination, pluripotency, embryonic stem cells, induced pluripotent stem cells

## Abstract

Pluripotent embryonic stem cells (ESCs) are derived from early embryos and can differentiate into any type of cells in living organisms. Induced pluripotent stem cells (iPSCs) resemble ESCs, both of which serve as excellent sources to study early embryonic development and realize cell replacement therapies for age-related degenerative diseases and other cell dysfunction-related illnesses. To achieve these valuable applications, comprehensively understanding of the mechanisms underlying pluripotency maintenance and acquisition is critical. Ubiquitination modifies proteins with Ubiquitin (Ub) at the post-translational level to monitor protein stability and activity. It is extensively involved in pluripotency-specific regulatory networks in ESCs and iPSCs. Ubiquitination is achieved by sequential actions of the Ub-activating enzyme E1, Ub-conjugating enzyme E2, and Ub ligase E3. Compared with E1s and E2s, E3s are most abundant, responsible for substrate selectivity and functional diversity. In this review, we focus on E3 ligases to discuss recent progresses in understanding how they regulate pluripotency and somatic cell reprogramming through ubiquitinating core ESC regulators.

In culture, embryonic stem cells (ESCs) can indefinitely self-renew while maintaining pluripotency [1]. These capabilities are attributed to the unique regulatory network that governs the ESC-specific gene expression profile and chromatin landscape. The factors anchored in the network include transcription factors (TFs), RNA molecules, and epigenetic regulators [2,3]. Particularly, Octamer-binding transcription factor-4 (Oct4), sex-determining region Y (SRY)-related high-mobility-group (HMG) Box 2 (Sox2), and Nanog form the core transcriptional regulatory circuitry governing pluripotency maintenance and acquisition [4,5,6]. How to maintain the precise levels of these core regulators is fundamental for their pivotal activity in ESC maintenance and pluripotency induction. Transcriptomic and proteomic analysis suggest extensive involvement of ubiquitination in pluripotency regulation [7,8,9,10]. In this review, we summarize the progress in understanding ubiquitination-mediated regulation of pluripotency maintenance and acquisition, and mainly discuss how E3s recognize and fine-tune the expressions and activity of core pluripotency regulators so as to achieve pluripotency maintenance and reinstatement.

## 1. Pluripotent Cells

**Figure 1:**
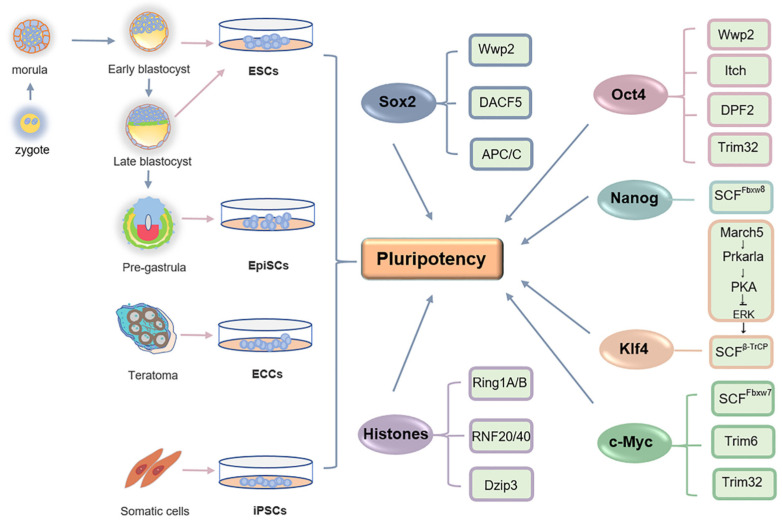
E3s are involved in regulating pluripotency. Pluripotent cells can be derived from teratomas or early embryos. Pluripotency can also be restored through introducing defined factors in somatic cells to generate iPSCs. Multiple E3s have been identified for their critical roles in monitoring the stability or activity of pluripotency regulators.

Embryonic development starts from the totipotent zygote. At the blastocyst stage, embryonic cells are diverged into the inner cell mass (ICM) and the external mono-layer trophectoderm [11]. The ICM cells are pluripotent since they can give rise to all three embryonic germ layers (ectoderm, endoderm, and mesoderm), but fail in forming a complete embryo alone [12]. Pluripotency is preserved by the epiblast cells that act as sources of all specialized progeny cells in living organisms [13]. Interestingly, the transient pluripotency of early embryonic cells can be represented by cultivated cells *in vitro* (Figure 1). Embryonal carcinoma (EC) cells are the first pluripotent cell type grown in culture. However, their application potential is limited because they are derived from teratomas/teratocarcinomas and usually abnormal in chromosomes [14,15,16,17,18,19,20,21,22]. The first “true” embryo-derived pluripotent cells are ESCs established by Martin Evans, Matthew Kaufman and Gail Martin [23,24]. ESCs can be isolated from the ICM or the epiblast of the mature blastocyst [23,24]. Interestingly, although embryos gradually lose pluripotency upon proceeding into the gastrulation stage, researchers isolate pluripotent epiblast stem cells (EpiSCs) from the post-implantation epiblast tissues [25,26]. Of note, ESCs and EpiSCs display two different pluripotent states. EpiSCs are primed pluripotent since they are deficient in chimera formation, though they can differentiate into all three germ lineages [26]. ESCs faithfully capture the naïve pluripotency resembling the in vivo ICM and epiblast of the blastocyst in the serum-free medium supplemented with two inhibitors (2i), PD0325901 and CHIR99021, respectively, inhibiting the Mek/Erk and Gsk3 activity [27]. They maintain superior differentiation capability and can consistently produce germline chimeras with normal diploid karyotype [4,12,13,28]. Thus, ESCs serve as an excellent model to study embryogenesis and perform cell replacement therapies. To date, ESCs have been successfully isolated and cultivated from various species, such as mice, humans, non-human primates, and pigs [23,29,30]. Embryonic origin of ESCs ineluctably brings about the ethical concerns surrounding ESC research. However, that has been alleviated by the seminal work of Takahashi and Yamanaka. The duo elegantly restored pluripotency through the introduction of defined factors in mouse somatic cells to generate induced pluripotent stem cells (iPSCs) [31]. Human iPSCs were derived soon afterwards [32,33,34]. iPSCs possess similar differentiation capacity with ESCs, and thus allow us to generate patient-specific pluripotent cells for cell therapy of degenerative and other human diseases. 

## 2. Ubiquitination

**Figure 2:**
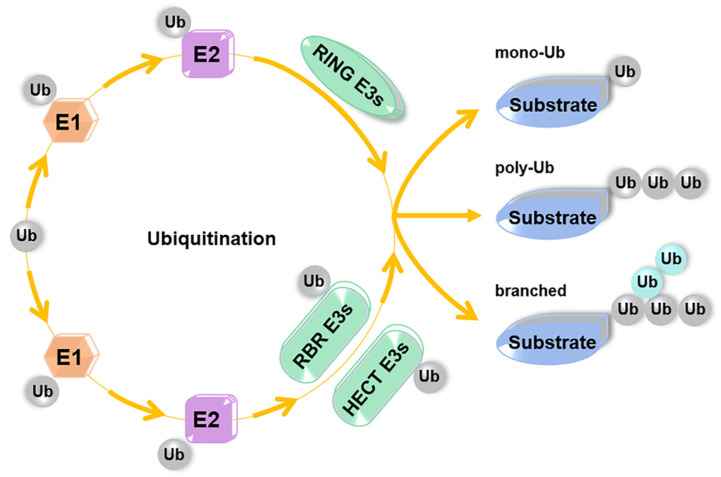
The process of ubiquitination. E1 activates Ub to form the E1-Ub intermediate. Next, E2 catalyzes transthiolation reaction to transfer Ub from the E1-Ub complex to its active C site. An E3 ligase is responsible for the final reaction. RING-type E3s facilitate direct Ub transfer from E2s to substrates, whereas HECT-type and RBR-type E3s adopt two-step reactions for Ub conjugation. Substrate proteins can be modified by mono-ub or poly-ub chains. A poly-Ub chain can be homotypic with only one linkage type or heterotypic with mixed or branched linkages.

Ubiquitination is a fundamental process post-translationally modifying proteins with a single Ub (monoubiquitination) or a poly-Ub chain (polyubiquitination) to regulate protein stability or activity [35,36]. Ub is a small-sized protein comprising only 76 amino acids and highly conserved among all types of eukaryotic cells [37,38,39]. In most cases, the C-terminal glycine 76 of Ub (Ub-G76) is conjugated to an internal lysine (K) residue of substrates via an isopeptide bond. However, some non-K residues of proteins, such as methionine-1 (M1), cysteine (C), serine (S), threonine (T) and tyrosine (Y), can serve as ubiquitination sites under some particular conditions [40,41,42,43,44]. To form a poly-Ub chain, the Ub-G76 of a distal Ub is covalently connected with one K residue of a proximal Ub. Ub harbors seven Ks in total, respectively, K6, K11, K27, K29, K33, K48, and K63. Any of these residues can be employed for poly-Ub chain assembly [45]. Alternatively, the G76 residue of the proximal Ub can connect with the M1 of the distal Ub to form a linear poly-Ub chain [46]. What is more, poly-Ub chains can be homotypic, or heterotypic (mixed or branched) comprising multiple linkage types, such as K11/K48-branched chains, K48/K63-branched chains, and K63/M1-linked hybrid linkage [47,48,49] (Figure 2).

In general, ubiquitination is achieved by three continuous catalytic reactions (Figure 2). The Ub-activating enzyme (Uba, E1) firstly initiates the conjugation process. It adenylates Ub-G76 in an ATP hydrolysis-dependent manner to form a Ub-adenosine monophosphate (Ub-AMP) intermediate. The intermediate subsequently accesses the E1 active cysteine (C) site via nucleophilic attack. A thioester bond is formed connecting the E1 with Ub-G76, accompanied with AMP release [50]. The second reaction is mediated by a Ub-conjugating enzyme (UBC, E2). E2 catalyzes transthiolation reaction, in which the Ub moiety is handed over from the E1-Ub complex to the active C site of its UBC domain. An E3 ligase is responsible for the final reaction. It simultaneously interacts with the E2-Ub and a protein substrate, and ligates Ub with the protein through mediating isopeptide bond formation. Compared with E1s and E2s, E3s are most abundant. According to sequence properties and Ub conjugation mechanisms, E3s can be classified into three major groups, including Really Interesting New Gene (RING) domain-containing E3s, Homologous to E6-AP COOH terminus (HECT) domain-containing E3s and RING between RING (RBR) domain-containing E3s. RING domain-containing E3 family members do not need to form any intermediate thioester linkage with Ub but facilitate direct Ub transfer from E2s to substrates. In this step, they simply act as a scaffold to bring the E2-Ub to the vicinity of the substrate so that the ε-amino group of the substrate K residue can attack the thioester linkage of E2-Ub to form a transient tetrahedral complex. The E2 is then released after it catalyzes transfer of Ub to the substrate [51,52]. The RING-type E3s constitute the largest E3 family, all of which contain a conserved C-rich RING finger motif or RING finger-like domain, such as the plant homeodomain/leukemia-associated protein (PHD/LAP) finger motif and U box [46,53,54,55]. Of note, the activity of the RING finger and PHD/LAP motif, rather than the U box, require chelation of Zn^2+^ ions [56]. Distinctive from the RING-type E3s, HECT domain-containing E3s and RBR domain-containing E3s contain intrinsic enzymatic C site and adopt two-step reactions for Ub conjugation. Firstly, they accept the E2-Ub at their active C site to form an E3-Ub thioester conjugate. Next, Ub is transferred from the E3 to the substrate [46]. Beside the C-terminal HECT domain, the HECT-type E3s generally harbor a variable N-terminal region responsible for specific substrate recognition [57]. According to sequence properties of N-terminal regions, the HECT-type E3s can be further grouped into three classes, respectively, the WW (two conserved tryptophans) domain-containing neuronal precursor cell-expressed developmentally downregulated 4 (Nedd4)/Nedd4-like E3s, HECT and regulator of chromosome condensation 1 (RCC1)-like domains (RLD)-containing (HERC) E3s, and other HECT-type E3s without WW and RLD domains [58]. Besides the WW domain, all Nedd4 family members harbor a C2 domain capable of binding with Ca^2+^ ions and phospholipids of intracellular membranes [59,60]. The WW domain is characterized by two conserved W residues separated by a 20–22 amino acid interval. It can capture the proteins harboring either proline (P)-rich motif or phosphorylation signals at S/T residues [61,62]. To date, nine Nedd4/Nedd4-like E3s have been identified in human cells, such as Smurfs and Wwp2 [63]. The HERC E3 group has six members. All of these ligases possess at least one RLD domain, which is characterized by seven repeats of 50–70 amino acids each [64]. The RBR-type E3s contain RING1 and RING2 domains [65]. The RING1 exhibit similar sequence properties with the canonical RING motif of RING-type E3s, and is responsible for binding with the E2-Ub intermediate. The RING2 domain contains a confirmed active C site mediating Ub conjugation through a similar two-step mechanism with the HECT-type E3s [66]. To date, 13 RBR E3s, such as Parkin, have been annotated in human, most of which are not well studied.

## 3. E3s-Mediated Regulation of Pluripotency Factors

### 3.1. Oct4

Oct4 belongs to the Pit-Oct-Unc (POU) transcription factor family. It contains three major modules, including the conserved DNA-binding POU domain in the middle separating the two transactivation domains, respectively, at the N and C terminus [67,68,69]. The POU domain comprises two individual fragments capable of independent DNA association, namely POU-specific domain (POUs) and POU homeodomain (POUh) [70]. Besides DNA association, POU can also mediate the protein-protein interaction of Oct4 with other factors [68]. Oct4 expression can be detected as early as the zygote and cleavage stage [71]. It is subsequently restricted to the ICM, embryonic ectoderm, and primordial germ cells (PGCs) [71,72,73,74,75]. *Oct4*-null zygotes can bypass the cleavage stage but stall at the blastocyst stage due to impaired ICM, suggesting its critical role in pluripotency regulation [76,77]. In ESCs, *Oct4* depletion impairs self-renewal and leads to cell differentiation into the trophectoderm lineage [1,78]. Strikingly, Oct4 acts as the most efficient reprogramming factor since its overexpression alone can successfully achieve iPSC formation [79,80]. However, excessive Oct4 is not favored by pluripotency maintenance. Two-fold induction of *Oct4* results in ESC differentiation into primitive endoderm and mesoderm [78]. Therefore, pluripotency requires an appropriate dosage of Oct4.

Wwp2 is the first identified E3 capable of mediating Oct4 ubiquitination. It belongs to the Nedd4 ligase family and contains four WW domains separating a N-terminal C2 domain from the HECT catalytic domain at the C terminus [81]. Wwp2 captures Oct4 for K63-linked polyubiquitination through the WW domain both in human and mouse ESCs [82,83]. Although it is not clear whether C2 domain-mediated Ca^2+^ and phospholipid binding is involved in this modification, the C2 domain is demonstrated required by the enzymatic activity of the HECT domain [83]. Wwp2-mediated ubiquitination suppresses not only the stability but transcriptional activity of Oct4 [82,83,84]. Furthermore, *WWP2* depletion increases the protein level of OCT4 in human ESCs [84]. However, this observation was not consistently reproduced by the study of Liao and Jin. They employed a similar strategy of short-hairpin RNA (shRNA) knockdown in human ESCs and found that OCT4 accumulation caused by *WWP2* depletion can only be observed in differentiated ESCs with retinoid acid (RA) treatment, but not in the unperturbed counterpart cells [83]. Moreover, *Wwp2*-null mouse ESCs remain undifferentiated with a normal protein level of Oct4 [85]. In vitro ubiquitination assay combining mass spectrometry (MS) analysis identified five putative loci targeted by Wwp2-mediated modification in Oct4, including K118, K121, K133, K137, and K144 [85]. Mutation of all these five K residues does not alter Oct4 stability in undifferentiated mouse ESCs, but markedly enhances the capability of Oct4 in reprogramming [85]. Consistently, *Wwp2* knockout increases the efficiency of Yamanaka factors-derived iPSC formation [85]. Therefore, Wwp2-mediated Oct4 ubiquitination appears dispensable for pluripotency maintenance but critical for cell fate determination and reprogramming.

The observation that *Wwp2* depletion has no impact on Oct4 stability in ESCs suggests that multiple E3s could be coordinated to monitor Oct4 stability for robust maintenance of pluripotency. Itch is another Nedd4 family E3 capable of modifying Oct4 [86]. Similar to Wwp2, it contains one N-terminal C2 domain, four WW domains, and the C-terminal HECT domain [87]. It is ubiquitously expressed in most types of mammalian cells and involved in regulating various biological processes, such as immune response, differentiation, and tumorigenesis [88]. In ESCs, Itch and Wwp2 can both modify Oct4 with K63-linked poly-Ub chain for 26S proteasome-mediated degradation, but do not share target residues [83,86]. The activity of Itch is inevitably required by pluripotency maintenance and acquisition since *Itch* depletion results in ESC differentiation and reduces the efficiency of iPSC formation [86]. At molecular level, although Itch-mediated ubiquitination promotes Oct4 degradation, this modification surprisingly enhances the affinity of Oct4 binding with target genes and thus increases its transcriptional activity [86]. Therefore, the transcriptionally activating effect by Itch could overwrite its degradative activity toward Oct4 in the process of pluripotency determination [86]. The Tripartite motif containing 32 (Trim32) belongs to the Trim-NHL family of RNA-binding proteins (RBPs) that are characterized by a three domain-containing module at the N terminus including one N-terminal RING enzymatic domain, at least one B-box domain, and one coiled-coil motif [89,90,91]. The C-terminal NHL domain mediates the binding of Trim32 with other proteins or microRNAs [92]. Trim32 can mediate ubiquitination of Oct4. However, this modification-induced Oct4 degradation displays in a RING domain-independent manner, indicating that Trim32 might indirectly regulate Oct4 stability [93]. Trim32 expression is increased after mouse ESCs differentiate, and its deletion enhances iPSC formation and abolishes LIF withdrawal- or RA treatment-induced iPSC differentiation [93]. PHD finger-containing E3 DPF2 is an interaction partner of Oct4 [94,95,96]. In human ESCs, it can assemble mixed polyubiquitin chains combining with K6, K48, and K63 linkages on OCT4, and promotes OCT4 proteasomal degradation [97]. Moreover, DPF2 appears involved in regulating the sub-nuclear localization of OCT4 [97]. However, whether the activity of DPF2 is required by pluripotency maintenance or iPSC formation is unknown.

### 3.2. Sox2

Sox2 contains a conserved HMG DNA-binding domain and a C-terminal transactivation domain [98,99]. In vivo, Sox2 can be detected in the zygote and its expression maintains throughout the blastomere, blastocyst, and gastrulation stage [100]. Distinctive from the other two core ESC regulators, Oct4 and Nanog, Sox2 is also expressed in the extraembryonic ectoderm, fetal neural lineages, and adult stem cells, such as neural stem cells, retina, and pituitary progenitors [100,101,102,103,104,105]. Sox2 is indispensable for early embryo development and *Sox2*-null embryos cannot survive due to failure in normal epiblast formation [100]. *Sox2* depletion in ESCs results in polyploidy formation and trophectoderm differentiation [106,107]. However, Sox2 elevation fails in reinforcing ESCs but leads to cell differentiation [106,107,108] As mentioned above, Oct4 similarly displays a dosage-dependent activity, implicating close relationship between these two factors. As expected, Sox2 and Oct4 always form a heterodimer to orchestrate the transcriptional regulatory network of ESCs [6,109]. Interestingly, the E3 of Oct4, Wwp2, is also able to ubiquitinate Sox2 for degradation [110]. Of note, ubiquitination closely crosstalks with methylation in the process of Sox2 modification. For instance, Wwp2-mediated Ub conjugation depends on a methylation signal at Sox2-K119 in mouse ESCs [110]. What is more, monomethylated Sox2 at the K42 and K117 residues can be captured by a methyl-binding protein L3MBTL3, which further recruits the RING-type E3 DCAF5 to ubiquitinate the methylated Sox2 for proteolysis [111]. Unmethylated Sox2 is ubiquitinated by the anaphase-promoting complex/cyclosome (APC/C) [112]. The APC/C is a large E3 complex comprising nearly twenty subunits [113]. Among these subunits, RING-type E3 Apc11 and Apc2 form the catalytic center, whereas Apc10 facilitates substrate recognition [114]. In mouse ESCs, APC/C cooperates with ubiquitin-conjugating enzyme E2s (Ube2s) to modify Sox2 with K11-linked poly-Ub chain at the K123 residue and thus destine it for 26S proteasome-mediated degradation [112]. In this process, Ube2s and APC10 are both responsible for Sox2 recognition. Interestingly, although APC/C-Ube2s promotes Sox2 degradation, *Ube2s* deletion does not drive ESCs to differentiate but reinforces the pluripotent state through inducing a set of pluripotency-related genes including *Esrrb, Nanog, Lin28a*, and *Sall4* [112]. These genes can replace other combinations of *Oct4*, *Sox2*, *Klf4*, and *c-Myc* to efficiently support pluripotency [115]. APC/C-Ube2s-mediated ubiquitination seems not dependent on methylation at Sox2-K42, K119, or K117 since any mutation of these loci does not abolish APC/C-Ube2s-mediated Ub conjugation to Sox2 [112].

### 3.3. Nanog

Nanog is encoded by *early embryo specific NK (ENK)* that was initially identified in mouse ESCs. Compared with other NK protein family members, it shares low sequence similarity and is exclusively involved in ESC maintenance and embryonic development [116,117,118]. Its unique function depends on multiple domains, respectively, the serine-rich N terminus, the DNA-binding homeodomain, and the tryptophan repeat (WR)-containing C terminus. The C terminus comprises three sub-regions, including the C-terminal activation domain 1 (CD1), the WR repeats in the middle, and the CD2 at the C-terminal end. The latter two sub-regions and the N terminus possess transactivation activity [119]. The expression of *Nanog* can be ubiquitously detected in all blastomeres, and is subsequently restricted to the ICM and the subsets of the epiblast cells including the primordial germ cells. It starts to decline during primitive streak formation [116,117,120]. *Nanog*-null embryos exhibit impaired ICM and die at 4.5 d.p.c due to failure in forming the primitive ectoderm [117]. Consistently, *Nanog* deletion results in ESC differentiation into endoderm lineages [116,117,121]. Conversely, *Nanog* overexpression confers ESCs resistance to exogenous induction of differentiation [116,117]. However, *Nanog* expression is not homozygous in ESCs under the serum culture condition, but stochastically fluctuates [122]. Although the *Nanog*-low ESCs can self-renew, they lose naïve pluripotency, failing in differentiation into germ lines [123]. K48- and K63-linked polyubiquitination are highly involved in monitoring the stability and activity of Nanog in ESCs [124]. To date, Skp1/Cul1/F-box (SCF)-like ligase complex SCF^F-box and WD40 domain-containing protein 8 (Fbxw8)^ is the only E3 identified mediating Nanog ubiquitination [125]. F-box protein Fbxw8 exerts substrate recognition [126,127,128,129]. It coordinates with RING finger-containing RING box protein-1 (Rbx1), bridging protein Skp1 and scaffold protein Culin 7 (Cul7) to assemble the multi-subunit SCF^Fbxw8^ complex [130]. In mouse ESCs, SCF^Fbxw8^-mediated ubiquitination of Nanog requires ERK1-activated phosphorylation signal. Moreover, *Fbxw8* depletion results in Nanog accumulation, consequently enhancing self-renewal and preventing ESCs from differentiation [125]. In vivo, *Fbxw8* deletion impairs embryo growth due to abnormal development of placenta [131,132]. However, it remains elusive whether Nanog acts as a dominant downstream effector of SCF^Fbxw8^ in the developmental process of embryos. Moreover, it will be of interest to uncover whether this ligase complex is involved in regulating naïve pluripotency or monitoring the fluctuating expression of Nanog in serum-cultured ESCs.

### 3.4. C-Myc

C-Myc oncoprotein belongs to the basic helix-loop-helix (bHLH) transcription factor superfamily, extensively involved in regulating cell metabolism, proliferation, differentiation, and apoptosis [133,134]. In mouse ESCs, c-Myc promotes self-renewal to support pluripotency and increase the efficiency of iPSC formation [31,135]. Although it preferentially binds with any open chromatin region without defined specificity for universal transcription activation, c-Myc possibly coordinates with Oct4, Sox2 and Nanog to selectively enhance transcription efficiency of ESC specific genes [136,137]. Of note, c-Myc acts distinctively in human ESCs since it fails in supporting self-renewal but promotes apoptosis and differentiation [138]. C-Myc harbors a short half-life rigorously regulated by ubiquitination [139,140]. The transactivation domain of c-Myc possesses two conserved Myc boxes (MBs), MB1 and MB2, providing binding loci for E3s [141,142]. Moreover, dual phosphorylation of MB1-T58/S62 residues promotes degradative ubiquitination of c-Myc, while S62 phosphorylation alone increases its stability instead [143,144]. Multiple E3s have been identified mediating ubiquitination of c-Myc, including SCF^Skp2^ ligase complex, SCF^Fbxw7^ complex, and TRIM6 [145,146,147,148]. F-box proteins, Fbxw7 and Skp2, recognize the MB1 and MB2, respectively, to mediate degradative ubiquitination [145,146,149]. Importantly, *Fbxw7*-null embryos die at E10.5. *Fbxw7* deletion results in c-Myc accumulation, which prevents mouse ESC differentiation [10]. Moreover, *Fbxw7* deletion promotes iPS cell formation, which, however, cannot be reversed by *c-Myc* depletion, indicating the existence of other substrates in the reprogramming process [10]. Another study by Egozi et al. explored the role of Skp2 in human ESCs. They demonstrated that high expression of Skp2 is co-related with the undifferentiated state of human ESCs. It selectively ubiquitinates cell cycle inhibitor p27, rather than c-Myc, for degradation to enhance proliferation and simultaneously prevent differentiation [150]. TRIM6 exerts important functions in pluripotency maintenance through ubiquitinating c-Myc independent of the phosphorylation signal at T58 and S62 residues [148]. Intriguingly, although *TRIM6*-depleted ESCs exhibit increased stability of c-Myc, TRIM6 elevation fails in reducing the stability of c-Myc but activates *Nanog* expression. Furthermore, *TRIM6* manipulation-resulted Nanog elevation could not confer ESC independence of LIF signal, which appears inconsistent with the previous finding that elevated Nanog inhibits LIF withdrawal-induced ESC differentiation [116,117]. Trim32 may also be involved in ubiquitinating c-Myc in pluripotent cells [93]. The interplay between these two factors has been explored in neural stem cells [151,152,153].

### 3.5. Krüppel-Like Factors (Klfs)

Klfs belong to the zinc finger-containing transcription factor family and are characterized by three krüppel-like C2H2 zinc finger DNA-binding domains. Although all Klfs have a similar DNA binding consensus, they function diversely through interacting with context-dependent partners via their variable N-terminal region [154]. There are eighteen Klf proteins widely involved in the regulation of cell proliferation, differentiation, migration, inflammation, metabolism, and apoptosis [155]. Among these Klfs, only Klf2, Klf4, and Klf5 are sensitive to ESC differentiation [1,156]. They form a core Klf transcriptional regulatory circuitry maintaining self-renewal and pluripotency [156,157,158]. Under the LIF/serum culture condition, single or dual depletion among *Klf2, Klf4*, or *Klf5* fails in driving ESC differentiation, but simultaneous triple knockdown of all these three genes results in loss of ESC identity [156]. However, under the 2i culture condition, naïve pluripotency of ESCs cannot be sustained after *Klf2* deletion [159]. Moreover, Klf4 activation is essential to reprogram primed pluripotent mouse EpiSCs or human ESCs into naïve pluripotent cells [160,161]. Due to the critical roles of Klf2 and Klf4 in maintaining naïve pluripotency, they are selected as “naïve” factors [160,162]. Similar to Oct4, Sox2, and Nanog, Klf2 and Klf5 exhibit very short half-lives in ESCs (about 3 h), which allow rapid response to any differentiation signals [163]. Interestingly, Klf4 is highly stable with a half-life of longer than 24 h, and this high stability is markedly decreased upon differentiation, which requires a mono-Ub signal at its K249 residue [163]. ERK1/2-mediated phosphorylation at the S123 residue can also reduce the stability of Klf4. The phosphorylation mark allows E3 SCF^β-TrCP^ to capture Klf4 for polyubiquitination and proteasomal degradation, which subsequently results in ESC differentiation [164]. On the other hand, Klf4 transcriptionally activate the expression of membrane-associated ring finger (C3HC4) 5 (March5) to sustain its high stability. March5 is a RING-type E3 and located in the mitochondria membrane, regulating pluripotency, senescence, reactive oxygen species (ROS) generation and mitochondrial division [165,166,167,168,169]. In mouse ESCs, March5 acts as an indispensable factor for pluripotency maintenance. Furthermore, it can coordinate the four Yamanaka factors (Oct4, Sox2. Klf4 and c-Myc) to increase the efficiency of iPSC formation [165]. At the molecular level, March5 polyubiquitinates cAMP-dependent protein kinase type I-alpha regulatory subunit (Prkar1a) to activate protein kinase A (PKA), which finally results in the inhibition of the ERK signaling pathway [165]. ERK inhibition stalls Klf4-S123 phosphorylation-induced degradative ubiquitination by SCF^β-TrCP^ and thus enhances Klf4 stability [164,165]. As expected, March5 can at least partially substitute for Klf4 to drive iPS cell formation [165].

### 3.6. Epigenetic Regulators

Pluripotent cells exhibit a unique epigenetic landscape, which is largely marked by histone modifications [170,171]. Compared with methylation and acetylation, information on correlation between histone ubiquitination and pluripotency is limited. Monoubiquitination of histone H2A-K119 and H2B-K120 has been relatively well documented. H2B-K120 monoubiquitination is mediated by a heterotetrameric E3 complex comprising Ring Finger Protein 20 (RNF20) and RNF40 [172]. This modification is always associated with transcription activation and is important for the differentiation process of ESCs, but not for ESC maintenance [173,174,175]. More recently, a study by Xie et al. demonstrated that RNF40 acts as a central mediator to promote pluripotency acquisition in the process of reprogramming [176]. Distinctive from H2B-K120 mono-Ub, H2A-K119 mono-Ub is correlated with transcription repression [177,178]. This signal can be mediated by RING-type E3 Ring1A/1B, and is critical for maintaining the undifferentiated state of ESCs. Double deletion of *Ring1A* and *Ring1B* results in ESC differentiation [179,180,181,182,183]. Another RING-type E3 Dzip3 can also mediate H2A-K119 monoubiquitination in ESCs and specifically acts at differentiation-related gene promoter regions through regulating 3D chromatin organization [184]. In addition to histone marks, a number of epigenetic regulators, such as Jarid2, Kdm3a, Hdac1, Dnmt3a, Jmjd1, Fbxo15, and Setdb1, display ubiquitination signals in ESCs [10,185]. However, the mechanisms underlying their ubiquitination and whether these signals are involved in regulating ESC identity remain elusive.

## 4. Concluding Remarks and Perspectives

Pluripotent ESCs and iPSCs exhibit unique transcriptomic, epigenomic, and proteomic signatures, which are precisely orchestrated by the interconnected regulatory network. Ubiquitination governs proteostasis and is one of the most abundant modification signals to monitor protein stability and activity. Therefore, ubiquitination is involved in nearly all protein-dependent cellular processes, including pluripotency maintenance and cell fate decision. In pluripotent ESCs, Ub/proteasome pathway-related genes are highly activated, and over one thousand proteins are marked by Ub signals [7,8,9,10]. Increasing studies identified pluripotency-related E3s through analyzing their interplay with core ESC regulators, such as Oct4, Sox2 and Nanog, and other pluripotency-related factors (Figure 1). For instance, ESC regulator Rex1 is ubiquitinated by E3 RNF12 for degradation, which activates X-chromosome inactivation of mouse ESCs [186]. Truncation mutation in RNF12 leads to loss of pluripotency and neuroectodermal differentiation [187]. These E3s appear tightly interconnected to form a pluripotency-specific ubiquitination network. For instance, Wwp2 can simultaneously ubiquitinate multiple substrates, Oct4, Sox2 and RNA polymerase II subunit B1 (Rpb1), in ESCs, which might efficiently reinforce the molecular signatures and network of pluripotency [81,84,110]. To further understand the ubiproteome in regulating pluripotency and reprogramming, questions remain to be investigated. For instance, how does ubiquitination dynamically regulate different states of pluripotency, such as naïve and primed states? How do E3 ligases coordinate with transcription factors and epigenetic regulators to prepare ESCs or iPSCs for an appropriate poised status during differentiation? Moreover, since ubiquitination always coexists with or requires other modification signals, how does it crosstalk with other types of modifications, such as methylation, acetylation, and phosphorylation, to globally orchestrate pluripotency-specific molecular signatures. Answers to these questions will provide greater insights into our understanding of ESCs and iPSCs, and further their applications in basic and clinical research of human diseases. One of the technical challenges is to develop antibodies or Ub-based chemical probes to capture any Ub linkage of interest with high specificity and sensitivity [188].

## Abbreviations

APC/C	Anaphase-promoting complex/cyclosome
bHLH	basic Helix-loop-helix
Cul7	Culin 7
EC	Embryonal carcinoma
ENK	Early embryo specific NK
EpiSCs	Epiblast stem cells
ESCs	Embryonic stem cells
Fbxw8	F-box and WD40 domain-containing protein 8
HECT	Homologous to E6-AP COOH terminus
HERC	HECT and regulator of RCC1-like domains-containing
HMG	High-mobility-group
ICM	Inner cell mass
iPSCs	induced Pluripotent Stem cells
Klfs	Krüppel-like factors
March5	Membrane-associated ring finger (C3HC4) 5
MBs	Myc boxes
MS	Mass spectrometry
Nedd4	Neuronal precursor cell-expressed developmentally downregulated 4
OCT4	Octamer-binding transcription factor-4
PGCs	Primordial germ cells
PHD/LAP	Plant homeodomain/leukemia-associated protein
PKA	Protein kinase A
POU	Pit-Oct-Unc
POUh	POU homeodomain
POUs	POU-specific domain
Prkar1a	Protein kinase type I-alpha
RA	Retinoid acid
RBPs	RNA-binding proteins
RBR	RING between RING
Rbx1	RING box protein-1
RCC1	Regulator of chromosome condensation 1
RING	Really Interesting New Gene
RLD	RCC1-like domains
RNF20	Ring Finger Protein 20
RNF40	Ring Finger Protein 40
ROS	Reactive oxygen species
Rpb1	RNA polymerase II subunit B1
SCF	Skp1/Cul1/F-box
shRNA	short-hairpin RNA
Sox2	SRY-related HMG Box 2
SRY	Sex-determining region Y
TFs	Transcription factors
TRIM32	Tripartite motif containing 32
Uba/E1	Ub-activating enzyme
Ub-AMP	Ub-adenosine monophosphate
UBC/E2	Ub-conjugating enzyme
Ube2s	Ubiquitin-conjugating enzyme E2s
Ub-G76	Glycine 76 of Ub
WR	tryptophan repeat
WW	two conserved tryptophans
